# Sulfur enhancement effects for uranium bioleaching in column reactors from a refractory uranium ore

**DOI:** 10.3389/fmicb.2023.1107649

**Published:** 2023-01-26

**Authors:** Qian Li, Yu Yang, Jinfang Ma, Jing Sun, Guangyue Li, Ruiyong Zhang, Zhao Cui, Ting Li, Xiaobei Liu

**Affiliations:** ^1^School of Resources and Environment and Safety Engineering, University of South China, Hengyang, China; ^2^Key Discipline Laboratory for National Defence of Biotechnology in Uranium Mining and Hydrometallurgy, University of South China, Hengyang, China; ^3^Key Laboratory of Marine Environmental Corrosion and Biofouling, Institute of Oceanology, Chinese Academy of Sciences, Qingdao, China; ^4^Open Studio for Marine Corrosion and Protection, Pilot National Laboratory for Marine Science and Technology (Qingdao), Qingdao, China

**Keywords:** uranium bioleaching, sulfur enhancement, uranium dissolution kinetics, bacterial community, mechanism model

## Abstract

The feasibility of sulfur enhancement for uranium bioleaching in column reactors was assessed with a designed mixed *Acidithiobacillus ferrooxidans, Acidithiobacillus thiooxidans* and *Leptospirillum ferriphilum* from a refractory uranium ore. The uranium extraction reached 86.2% with the sulfur enhancement (1 g/kg) in 77 days leaching process, increased by 12.6% vs. the control without sulfur addition. The kinetic analysis showed that uranium bioleaching with sulfur enhancement in columns followed an internal diffusion through the product layer-controlled model. Ore residue characteristics indicated that sulfur enhancement could strengthen the porosity of passivation layer, improving the ore permeability. Notably, bacterial community analysis showed that sulfur enhancement at 1 g/kg could make the iron-oxidizing and sulfur-oxidizing bacteria on the ore surface maintain a good balance (approx. 1:1), and thus decomposing ore more effectively. Lastly, a possible mechanism model for uranium bioleaching with sulfur enhancement was proposed.

## 1. Introduction

Uranium has been increasingly applied in the electricity generation and defense industry as an important source of nuclear fuel. The increasing application of uranium has become a severe challenge for uranium resource recovery due to the decline of high-grade ore reserves available for mining and extraction using conventional technologies (Mudd, 2014). Moreover, the conventional chemical technologies for mineral processing are high-cost and ecologically unfriendly in the processing of the low-grade uranium-bearing ores, especially these associated with the refractory minerals like brannerite and coffinite (Abhilash and Pandey, 2013a; Bhargava et al., 2015).

Bioleaching has been extensively applied in the extraction of metals (e.g., copper and gold) from low-grade ores (Klaus, 1997; Rawlings, 2002; Abhilash and Pandey, 2013a; Srichandan et al., 2019; Wang et al., 2019; Kaksonen et al., 2020; Roberto and Schippers, 2022). It has been proved to be an effective approach to extract uranium from low-grade or complex refractory ores because of its economic feasible and environmentally sustainable (Tuovinen and Bhatti, 1999; Abhilash and Pandey, 2013a; Wang et al., 2019; Kaksonen et al., 2020). Bioleaching microorganisms play a critical role in the oxidative decomposition of many sulfide ores (Liao et al., 2020; Yin et al., 2020). Chemoautotrophic leaching bacteria can gain ATP by the oxidation of ferrous iron and/or reduced inorganic sulfur compounds (Vera et al., 2013; Ma et al., 2017). It was demonstrated that mixed iron-oxidizing bacteria and sulfur-oxidizing bacteria decompose minerals more effectively when presented as symbiotic consortia in bioleaching operations (Brune and Bayer, 2012; Li et al., 2017).

Recently, it was found that an appropriate Fe/S ratio in the ores is critical for the growth and activities of the bioleaching microorganisms, which would be the prerequisite for the synergistic effects of the bacterial consortia (Xia et al., 2009). The process of community succession and metabolism of a microbial consortium composed of *Acidithiobacillus thiooxidans* (*A. thiooxidans*), *Acidithiobacillus ferrooxidans* (*A. ferrooxidans*) and *Leptospirillum ferriphilum* (*L. ferriphilum*) were closely related to the leaching behavior of minerals, and could be regulated by mineral composition and element ratio like iron/sulfur ratio (Yang et al., 2021; Sun et al., 2022; Wu et al., 2022). The variation in energy metabolism structure of the microbial community during chalcopyrite bioleaching with different iron/sulfur ratios was proved different (Yang et al., 2021). Optimizing the energy metabolism structure of microbial community by adjusting the iron/sulfur ratio may be the key for improvement of the bioleaching (Feng et al., 2015). It was reported that addition of sulfur could increase the diversity of the bioleaching community, and an improved copper dissolution (~6%) was reached with the addition of 3.193 g/L sulfur (Xia et al., 2012). Uranium ores are generally oxide ores, which are almost absent of reduced inorganic sulfur compounds (Lottering et al., 2007; Dorota et al., 2015). Therefore, the exploration on sulfur enhancement of uranium bioleaching is critical and essential to recover uranium from the complex and refractory ores.

*A. ferrooxidans, A. thiooxidans* and *L. ferriphilum* are three typical mesophiles in bioleaching. The iron-oxidizers like *L. ferriphilum* can generate ferric iron to dissolve metal minerals, but this would easily lead to the accumulation of jarosite on the mineral surface (Vera et al., 2013). The sulfur-oxidizers like *A. thiooxidans* could oxidize a variety of sulfur compounds, including thiosulfate, sulfur, sulfite and sulfide (Vera et al., 2013; Yin et al., 2019). A third group like *A. ferrooxidans* can oxidize both sulfur and iron as its energy source (Vera et al., 2013). The electrons generated from the oxidation of elemental sulfur and/or reduced inorganic sulfur compounds would be transferred via the quinone pool (QH2) in the inner membrane directly to terminal oxidases or a periplasmic high potential iron-sulfur protein (HiPIP) in *A. ferrooxidans* or via other pathway (Amouric et al., 2011).

Column leaching generally aims at simulating the industrial applications, e.g., heap or dump leaching processes. Column leaching at laboratory scale can give valuable information on what has to be expected in heap or dump leaching and how the leaching operations to be optimized (Qiu et al., 2011; Srichandan et al., 2020). The purpose of this study was to evaluate the feasibility of sulfur enhancement for uranium bioleaching in column reactors from a complex and refractory uranium ore by an acidophilic consortium consisting of *A. ferrooxidans, A. thiooxidans* and *L. ferriphilum*. The performance of uranium bioleaching upon sulfur enhancement was investigated in a column reactor system. The uranium dissolution kinetics and microbial succession in both planktonic and biofilm phases were analyzed. Lastly, a model for the enhanced uranium bioleaching with sulfur enhancement was discussed based on the solution chemistry, bacterial community evolution and leaching behaviors.

## 2. Materials and methods

### 2.1. Ore preparation and characteristics

The uranium-bearing ore used in the experiment was collected from a granite uranium deposit in Guangdong Province, China. The ore sample was crushed and sieved to obtain five size fractions: 3–5 mm (26.81%), 1–3 mm (36.28%), 0.5–1 mm (15.96%), 0.1–0.5 mm (16.37%), <0.1 mm (4.57%). A representative sample was prepared by coning and quartering for mineral phase and chemical analysis. The mineral phase was analyzed by Mineral Liberation Analysis (MLA250, FEI, Czech). The MLA analysis showed that the main mineral was granite porphyry, and the main uranium-bearing minerals were brannerite (0.21%), coffinite (0.15%) and uraninite (0.02%). It was a refractory and complex uranium ore. Other metalliferous minerals included goethite 1.59%, pyrite 0.05%, zircon 0.08%, Rutile 0.25%, etc. The main gangue minerals were albite (69.72%), quartz (4.18%), apatite (15.65%), almandine (2.82%), muscovite (1.81%), jadeite (1.71%), calcite (0.83%), etc. The uranium grade of the ore sample analyzed by ammonium vanadate titration was 0.24%. The other chemical components analyzed by X-ray fluorescence spectrometer (XRF) showed SiO_2_ 55.11%, SO_3_ 0.17%, CaO 8.44%, Al_2_O_3_ 18.08%, Fe_2_O_3_ 2.79%, Na_2_O 8.77%, P_2_O_5_ 4.40%, K_2_O 0.34%, MgO 0.91%, TiO_2_ 0.26%, ZrO_2_ 0.06%, ZnO 0.03%, MnO 0.08%.

### 2.2. Bacterial strains and cultivation

The bacterial strains *A. ferrooxidans* ATCC 23270*, A. thiooxidans* A01 and *L. ferriphilum* YSK were selected to construct a bioleaching consortium for bioleaching experiments. *A. ferrooxidans* ATCC 23270 (Selkov et al., 2000) was obtained from American type culture collection (ATCC). *A. thiooxidans* A01 (Yin et al., 2014) was isolated from a coal heap drainage in Pingxiang, Jiangxi province, China, while *L. ferriphilum* YSK (Gao et al., 2007) was isolated from a drainage in Dexing copper mine in Jiangxi province, China. The leaching organisms were activated by inoculating in 100 mL culture medium to an initial density of ~5 × 10^5^ cells/mL (determined by hemocytometry) in 250-mL Erlenmeyer flasks. The medium was the 0K basal salt medium [(NH_4_)_2_ SO_4_ 3 g/L, KCl 0.1 g/L, K_2_HPO_4_ 0.5 g/L, MgSO_4_·7H_2_O 0.5 g/L, Ca (NO_3_)_2_ 0.01 g/L, pH 2.2 adjusted with H_2_SO_4_, sterilized at 120 °C for 20 min] (Selkov et al., 2000; Li et al., 2017). 44.7 g/L FeSO_4_·7H_2_O were added for *A. ferrooxidans* ATCC 23270 and *L. ferriphilum* YSK as energy source, while 10 g/L elemental sulfur for *A. thiooxidans* A01. Cells of *A. ferrooxidans* ATCC 23270 and *A. thiooxidans* A01 were cultivated at 30°C, while *L. ferriphilum* YSK at 40 °C aerobically with 180 rpm in an incubator shaker.

The activated strains were subjected to serial adaptation in the leach liquor adsorbed with resin (resin adsorption tail liquor) and with 24.8 g/L FeSO_4_·7H_2_O for *A. ferrooxidans* and *L. ferriphilum* or 5 g/L S^0^ for *A. thiooxidans* until a constant iron oxidation rate or growth rate was achieved. Each adapted strain was scale-up cultivated in an aeration tank, and then equally mixed for subsequent column bioleaching.

### 2.3. Column leaching experiments

**Figure 1:**
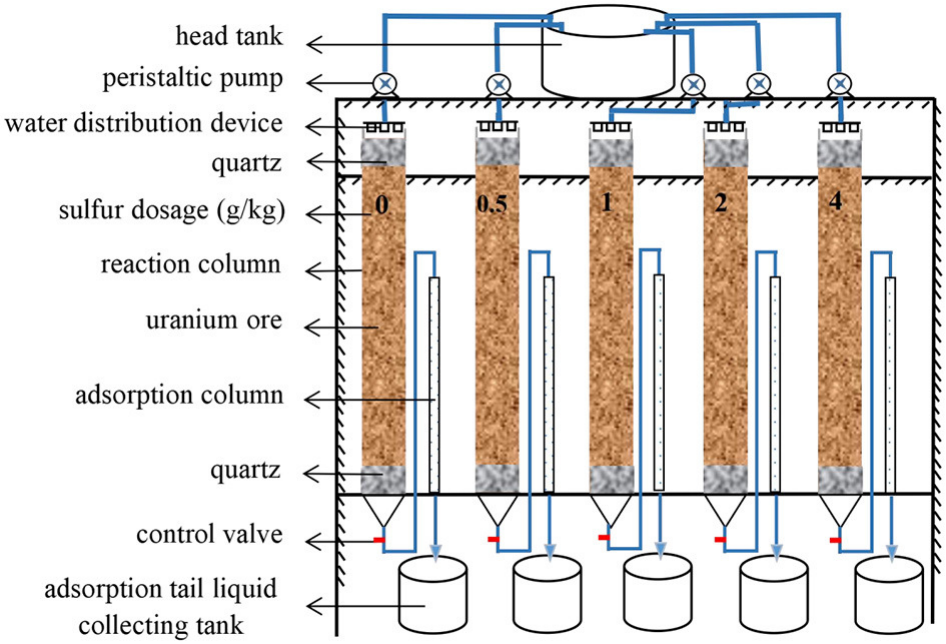
Schematic of column bioleaching devices.

To assess the effect of sulfur enhancement for uranium bioleaching in column reactors, four groups with sulfur dosages of 0.5, 1, 2, and 4 g/kg ore were designed, while no sulfur addition as blank control. The schematic of column bioleaching reactors is shown in Figure 1.

The columns were fabricated from acrylic tubes with a 10 mm-thick-wall. These reaction columns were 100 cm in height and 5 cm in internal diameter. The adsorption columns were 70 cm high with an internal diameter of 1 cm and connected to each reaction column. The column leaching experiments included two phases, acidic pre-leaching and microbial leaching (Yang et al., 2022). The column leaching turned into bioleaching phase when pH of the leaching liquor was at approx. 3.0. Bioleaching microorganisms of each strain were individually inoculated at 20% (v/v) to get a cell density of approx. 10^8^ cells/mL. The oxidation rate of ferrous iron reached 90%.

pH, redox potential, levels of Fe^2+^, Fe^3+^ and uranyl ions in feed solution (input) and leach liquor (output solution) were measured each day. The experiment was terminated when the concentration of uranyl ions in the leach liquor was less than 20 mg/L. The ore residues were sampled and analyzed for surface characterization and uranium quantification. The genomic DNA of the planktonic microorganisms and the attached microorganisms were extracted for microbial community analysis.

### 2.4. Analysis methods

The pH value was measured using a pH meter (PHBJ-260, INESA, China); the redox potential was measured by a platinum electrode with an Ag/AgCl reference electrode; total iron concentration in solution was assayed by titration with EDTA; the Fe^2+^ concentration was detected by titration with potassium dichromate (K_2_Cr_2_O_7_) (Nemati and Harrison, 2000). The surface morphologies of the original ore and leaching residues were characterized by scanning electron microscopy (SEM) (Sigma300, Carl Zeiss AG, Germany). The chemical components of the raw ore and residues were analyzed using X-ray fluorescence spectrometer (XRF) (S4 pioneer, Bruker, Germany).

The uranium concentration in solution and solid phase was titrated volumetrically with the ammonium vanadate method (Furman et al., 1951). The solid samples for titration analysis were first ground, and then the powered samples were boiled in perchloric acid till dried up for digesting the organics. Subsequently, the boiled samples were dissolved in a mixed solution of 10 mL hydrochloric acid, 3 mL hydrogen peroxide (30%) and 1 mL hydrofluoric acid. The filtrate was used for titration analysis (Sun et al., 2020).

For DNA extraction from the planktonic microorganisms, 150 mL solution sample were centrifuged at 10,000 g for 10 min to pellet the cells. Attached cells from the ore surfaces were collected from ore residues by repeated vortex and elution. Briefly, 10 g ore samples mixed with 4 g glass beads (diameter of 0.5 mm) and 50 mL sterile water were put in a 250 mL centrifuge bottle. They were vortexed in a rotary shaker at 220 rpm for 10 min. Afterwards, the mixture was centrifuged at 2,000 g for 2 min to separate the ore residue from the solution. The separated solution was centrifuged at 10,000 g for 10 min to pellet the cells. The supernatant was used to wash the ore residue circularly. The procedure was repeated for five times to collect effectively the attached microorganisms. Both the genomic DNA of the attached microorganisms and planktonic cells was extracted using the TIANamp^®^ Bacteria DNA kit (Tiangen Biotech Co. Ltd., Beijing, China) (Li et al., 2017). The DNA samples were checked on 1% agarose gel, and DNA concentration and purity were determined with a NanoDrop 2000 UV-vis spectrophotometer (Thermo Scientific, Wilmington, USA). The hypervariable region V3–V4 of the bacterial 16S rRNA gene was amplified with the primer pairs 338F (5′-ACTCCTACGGGAGGC AGCAG-3′) and 806R (5′-GGACTACHVGGGTWTCTAAT-3′) in an ABI GeneAmp 9700 PCR thermocycler (ABI, CA, USA) as described previously by Wang et al. (2018). Purified amplicons were pooled in equimolar and paired-end sequenced on an Illumina MiSeq PE300 platform/NovaSeq PE250 platform (Illumina, San Diego, USA) according to the standard protocols by Majorbio Bio-Pharm Technology Co. Ltd. (Shanghai, China) (Wang et al., 2018).

## 3. Results and discussion

### 3.1. Uranium mineralogy and feasibility analysis of sulfur enhancement in bioleaching

The mineralogy analysis by MLA showed that the uranium grade of the ore sample was 0.24%, and the uranium-bearing minerals were mainly brannerite [UO_2_·(TiO_2_)_2_], coffinite [UO_2_·(SiO_2_)_0.9_·(H_2_O)_0.2_] and uraninite (UO_2_). Uranium was tetravalent in the ore, which was hardly soluble in the aqueous solution. Thus, the ore sample was a complex and refractory uranium ore, and the addition of oxidizing agent was necessary to oxidize the tetravalent uranium to hexavalent uranium for dissolution purpose. XRF analysis showed that the iron content (2.79%) of the raw ore was sufficient for leaching of uranium. However, it would be transferred into jarosite on the surface of ore particles if only using iron-oxidizers like *L. ferriphilum*, or using iron/sulfur-oxidizers like *A. ferrooxidans* without enough sulfur as growth substrate. When using the single microbial species or single energy substrate, it would lead to the passivation accumulation on the mineral surface, which acts as a barrier against the diffusion of ions and then inhibits the uranium dissolution (Li et al., 2017). Herein, mixed iron-oxidizing bacteria and sulfur-oxidizing bacteria assist metabolic activities cooperatively and decompose minerals more effectively when presented at an appropriate Fe/S ratio in bioleaching operations. The mineral components analysis by XRF showed that the total sulfur content in the ore sample was 0.17%, indicating that sulfur enhancement for mixotrophic bioleaching was one of necessary and feasible ways in this study.

### 3.2. Effects of sulfur enhancement on pH and redox potential

**Figure 2:**
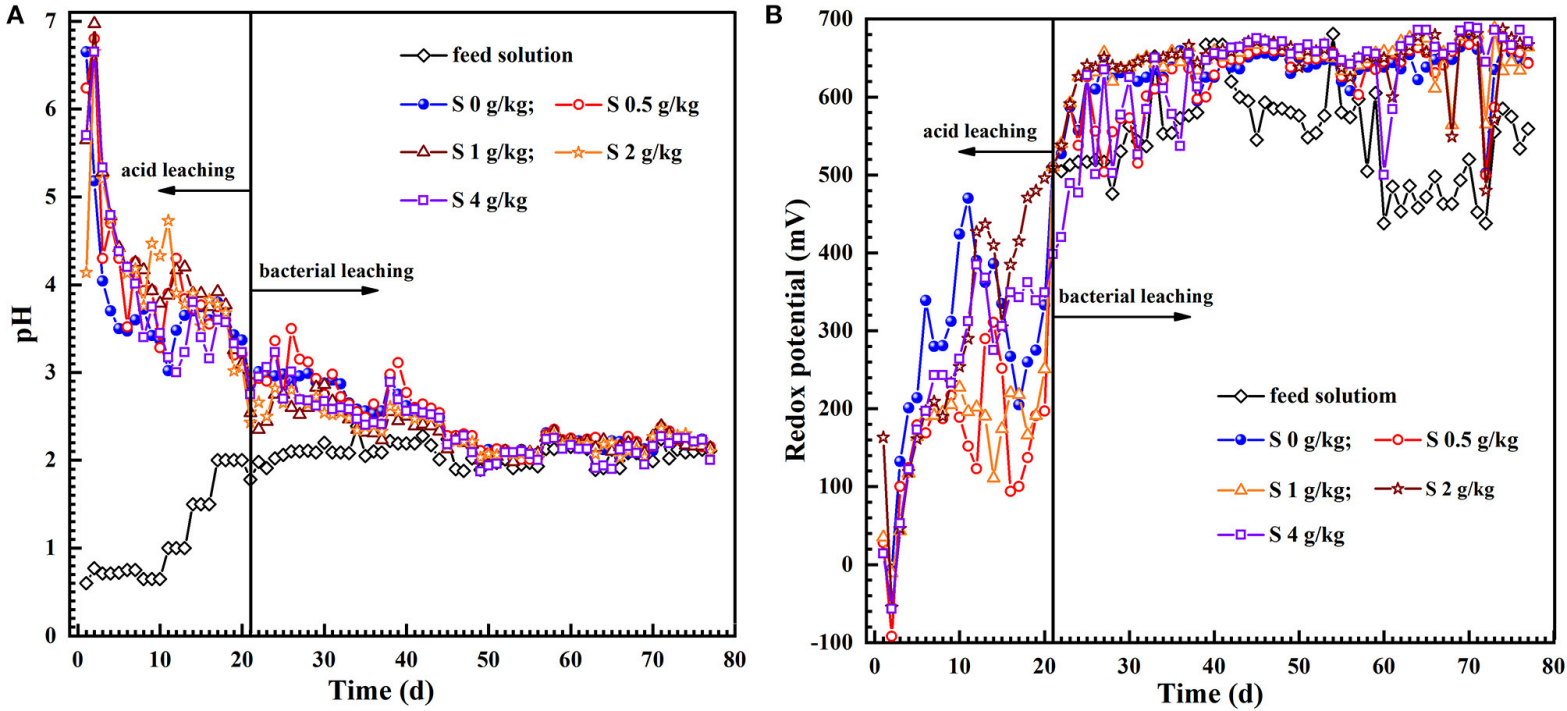
The dynamics of the pH **(A)** and redox potential **(B)** in the feed solution and leach liquor during uranium bioleaching in the column reactors with different sulfur dosages (0, 0.5, 1, 2, and 4 g/kg).

Figure 2A shows that the pH of leach liquors in the initial 5 days in the acidizing phase ascended very fast but the solution pH was still <7.0. This phenomenon was ascribed to the newly exposed acid-consuming minerals that consumed a large amount of H^+^ (Ram et al., 2020), resulting in a sharp rise in the pH of the leachate. Expectedly, the pH of each group gradually decreased with the proceeding of acidification, and bioleaching stage started by inoculation after 21 days acid pre-leaching. In the initial 13 days of bioleaching stage, the pH of the leach liquor was slightly lower in the tests with sulfur dosages of 1, 2 and 4 g/kg than those of 0.5 g/kg or absent of sulfur. Sulfur can be as energy substance for the growth of *A. thiooxidans* and *A. ferrooxidans*, which is conducive to acid production [Reaction (1)]. Subsequently, the variation trend of pH slightly fluctuated and was relatively stable in the late-bioleaching phase. It was likely attributed to that the protons could be consumed owing to the Fe^2+^ oxidation by *L. ferriphilum* and *A. ferrooxidans* simultaneously, resulting in a slight increase of pH at intervals in the bioleaching process [Reaction (2)]. Although the pH deviations of the leach liquor with different sulfur enhancement in each group were tiny in the later stage of bioleaching, it can still be seen that the pH value was a little lower than the control absent of sulfur (Figure 2A).


(1) $$\begin{array}{l} 2\text{S}+3{\text{O}}_{2}+2H_{2}O\;\underset{\to }{A.thiooxidans/A.ferrooxidans}\;4H^{+}+2SO_{4}^{2-} \end{array}$$

(2) $$\begin{array}{l} 4\text{F}{\text{e}}^{2+}+O_{2}+4H^{+}\;\underset{\to }{L.ferriphilum/A.ferrooxidans}\;4Fe^{3+}+2H_{2}O \end{array}$$

**Figure 3:**
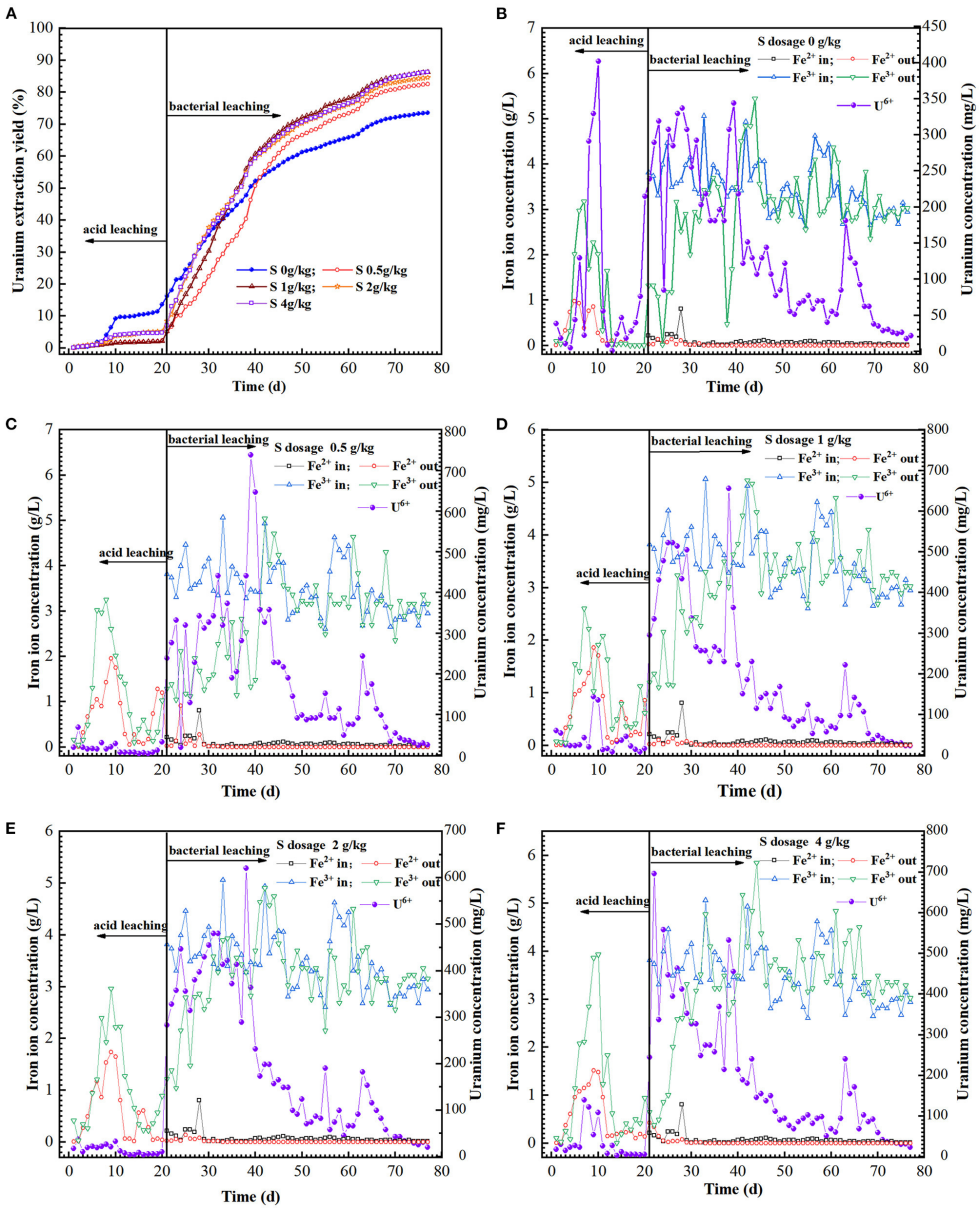
Variation of uranium extraction yield **(A)**, UO${}_{2}^{2+}$, Fe^2+^ and Fe^3+^ ions concentration **(B–F)** in feed solution (in) and leach liquor (out) as a function of time in the column reactors with different sulfur dosages (0, 0.5, 1, 2, and 4 g/kg).

The uranium extraction could be achieved at low pH and high redox potential (Qiu et al., 2011; Tavakoli et al., 2017). The variation in redox potential of the solution is mainly determined by the presence of iron as the Fe^3+^ or Fe^3+^/Fe^2+^ ratio in the leaching solution (Yue et al., 2014). The redox potentials in the acid pre-leaching phase were all less than 450 mV (Figure 2B), and the reaction between the leaching solution and some acid-consuming substances in the minerals were the main reactions during this phase, and the U (VI) in the ore was dissolved by H^+^ attack [Reaction (3)]. After inoculation, the level of ferric iron and redox potential of the leach liquor increased exponentially with time (Figures 2B, 3B–F). Unexpectedly, the redox potential of the leach liquor was a little lower at the sulfur dosage of 0.5 g/kg. Furthermore, the uranium dissolution also ascended rapidly (Figures 3C–F). Afterwards, the redox potential kept a little higher than 650 mV and remained relatively stable in the assays with 1–4 g/kg sulfur dosages.


(3) $$\begin{array}{l} \text{U}{\text{O}}_{3}+2H^{+}\overset{}{\underset{}{\to }}UO_{2}^{2+}+H_{2}O \end{array}$$

### 3.3. Effects of sulfur enhancement on uranium dissolution

The hexavalent uranium in the ore had been dissolved in the acid pre-leaching phase [Reaction (3)], while the tetravalent uranium is hardly soluble in the aqueous solution. In the acid pre-leaching stage, the uranium extraction with sulfur addition was approx. Ten percent lower than the control (Figure 3A). It was possibly ascribed into that the sulfur powder in the reaction column formed into passivation layer on the ore surface in acid pre-leaching stage, which would inhibit the ion diffusion and uranium dissolution to some extent (Pathak et al., 2017).

The sulfur-oxidizer *A. thiooxidans* can rapidly oxidize the sulfur layer on the ore surface (He et al., 2014; Kim et al., 2021) and reduce the pH in the leaching system, which was conducive to uranium bioleaching. Fe^3+^ concentration gradually increased due to the oxidation capability of *A. ferrooxidans and L. ferriphilum* (Figures 3B–F). The uranium concentration in the leach liquor was 600 mg/L with sulfur enhancement (0.5–4 g/kg), while it was less than 350 mg/L for the control. The Fe^3+^ generated by iron-oxidizers can oxidize U (IV) to UO${}_{2}^{2+}$ by an indirect mode [Reaction (4)] as described previously (Tributsch, 2001; Qiu et al., 2011). Extracellular polymeric substances (EPS) secreted by the bacteria can enrich Fe^3+^ and form EPS-Fe^3+^ complex to increase the oxidation efficiency of U (IV) to UO${}_{2}^{2+}$, namely indirect-contact mode [Reaction (5)] (Tributsch, 2001; Sand et al., 2001; Yu et al., 2011). Furthermore, the SO${}_{4}^{2-}$ generated by *A. thiooxidans* could have complexation reaction with UO${}_{2}^{2+}$ in the leachate under acidic conditions (pH ≤ 4.5) [Reaction (6)] (Vercouter et al., 2008; Abhilash and Pandey, 2013b), which can promote the uranium dissolution kinetics. After 77 days of leaching, the total uranium extraction of the assays with a sulfur dosage of 0, 0.5, 1, 2, and 4 g/kg were 73.6, 82.5, 86.2, 84.5, and 86.3%, respectively (Figure 3A). The results indicated that the gross uranium extraction could be increased by approx. 12.6% with appropriate sulfur enhancement.


(4) $$\begin{array}{l} \text{U}{\text{O}}_{2}+2\text{F}{\text{e}}^{3+}\overset{}{\underset{}{\to }}UO_{2}^{2+}+2Fe^{2+} \end{array}$$

(5) $$\begin{array}{l} \text{U}{\text{O}}_{2}+2\left(\text{EPS}-\text{F}{\text{e}}^{3+}\right)\overset{}{\underset{}{\to }}UO_{2}^{2+}+2\left(EPS-Fe^{2+}\right) \end{array}$$

(6) $$UO_{2}^{2+}+nSO_{4}^{2-}\to UO_{2}{\left(SO_{4}\right)}_{n}^{2-2n}$$

To find clearly out the differences of the sulfur enhancement in the bioleaching phase, the uranium extraction yield in acid pre-leaching can be neglected (the same starting point as average extraction yield in the acid pre-leaching period) (Supplementary Figure 1). Thus, the uranium extraction was 35.06% with sulfur enhancement of 1–4 g/kg in the initial 13 days, which was increased by approx. 11% vs. that of 0.5 g/kg sulfur or in the absence of sulfur. Notably, the uranium extraction with sulfur enhancement increased by approx. 20%, compared to these in the absence of sulfur. However, the excessive increase in sulfur to some extent inhibited the uranium dissolution, and thus, it maintained the highest at the sulfur dosage of 1 g/kg. After 27 days of bacterial oxidation, the uranium extraction with 0.5 g/kg sulfur was also higher than that of 2 g/kg. It showed that the uranium leaching was positively proportional to the sulfur dosage in the first 13 days of bioleaching. Afterwards, the leaching of the assays with sulfur addition of 0.5 and 1 g/kg was faster than that with 2 and 4 g/kg.

Furthermore, in order to evaluate the uranium extraction rate, the uranium dissolution kinetics in column bioleaching process was analyzed. The bulk or granular ores was subjected in the column bioleaching reactors, which is a typical dynamic process of liquid-solid multiphase reaction. If the uranium ore particles are regarded as spherical, the leaching kinetics of uranium ore can be described by the shrink kernel model (SCM). In this model, the uranium dissolution rate can be dependent on the following rate-limiting steps, which is the surface chemical reactions controlled kinetic model (Eq 1) or the internal diffusion through product layer controlled kinetic model (Eq 2) (Abdel-Aal, 2000; Sun et al., 2017).


(7) $$\begin{array}{l} 1-{\left(1-x\right)}^{\frac{1}{3}}=k_{1}t \end{array}$$

(8) $$\begin{array}{l} 1-\frac{2}{3}{x-\left(1-x\right)}^{\frac{2}{3}}=k_{2}t \end{array}$$

Where, *t* is the reaction time (d); *k*_1_ is the chemical reaction rate constant; *k*_2_ is the diffusion rate constant, *x* is the fraction of uranium extraction.

**Figure 4:**
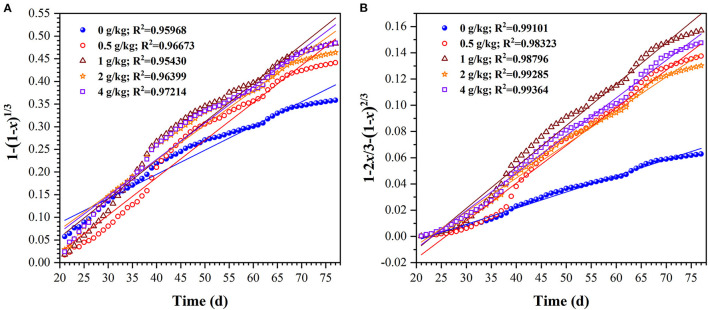
Plots of 1-(1-*x*)^1/3^ **(A)** and 1–2*x*/3-(1–*x*)^2/3^ **(B)** for uranium bioleaching as a function of time in column reactors with different sulfur dosages (0, 0.5, 1, 2, and 4 g/kg).

To determine the uranium rate-limiting step, Eqs 7, 8 were used to fit the experimental data, and the fitting degree was evaluated by correlation coefficient (R^2^) values. The results of each model are plotted in Figure 4. The apparent reaction rate constants (*k*_1_ and *k*_2_) and correlation coefficients (R^2^) for the two model above are given in Table 1. Figure 4 and Table 1 showed that the R^2^ fitted by the second model was >0.98. This indicates that the internal diffusion through the product layer-controlled model was more applicable to the uranium column bioleaching process with sulfur enhancement. Besides, it also showed that appropriate sulfur dosages could increase the chemical reaction rate (Figure 4A). However, the excessive sulfur was likely to inhibit the diffusion rate owing to the formation of a passivation layer.

**Table 1:** Reaction rate constants and correlation coefficients for two kinetic leaching models.

**Sulfur dosage (g/kg)**	**1 – (1 –** ***x*****)**^**1/3**^		**1 – 2*****x*****/3 – (1 –** ***x*****)**^**2/3**^	
	***k**_1_* **/min** ^−1^	**R** ^2^	***k**_2_* **/min** ^−1^	**R** ^2^
0	5.34 × 10^−3^	0.95968	1.23 × 10^−3^	0.99101
0.5	8.27 × 10^−3^	0.96673	2.87 × 10^−3^	0.98323
1	8.49 × 10^−3^	0.95430	3.16 × 10^−3^	0.98796
2	7.68 × 10^−3^	0.96399	2.57 × 10^−3^	0.99285
4	8.04 × 10^−3^	0.97214	2.87 × 10^−3^	0.99364

### 3.4. Effects of sulfur enhancement on ore residue characteristics

**Figure 5:**
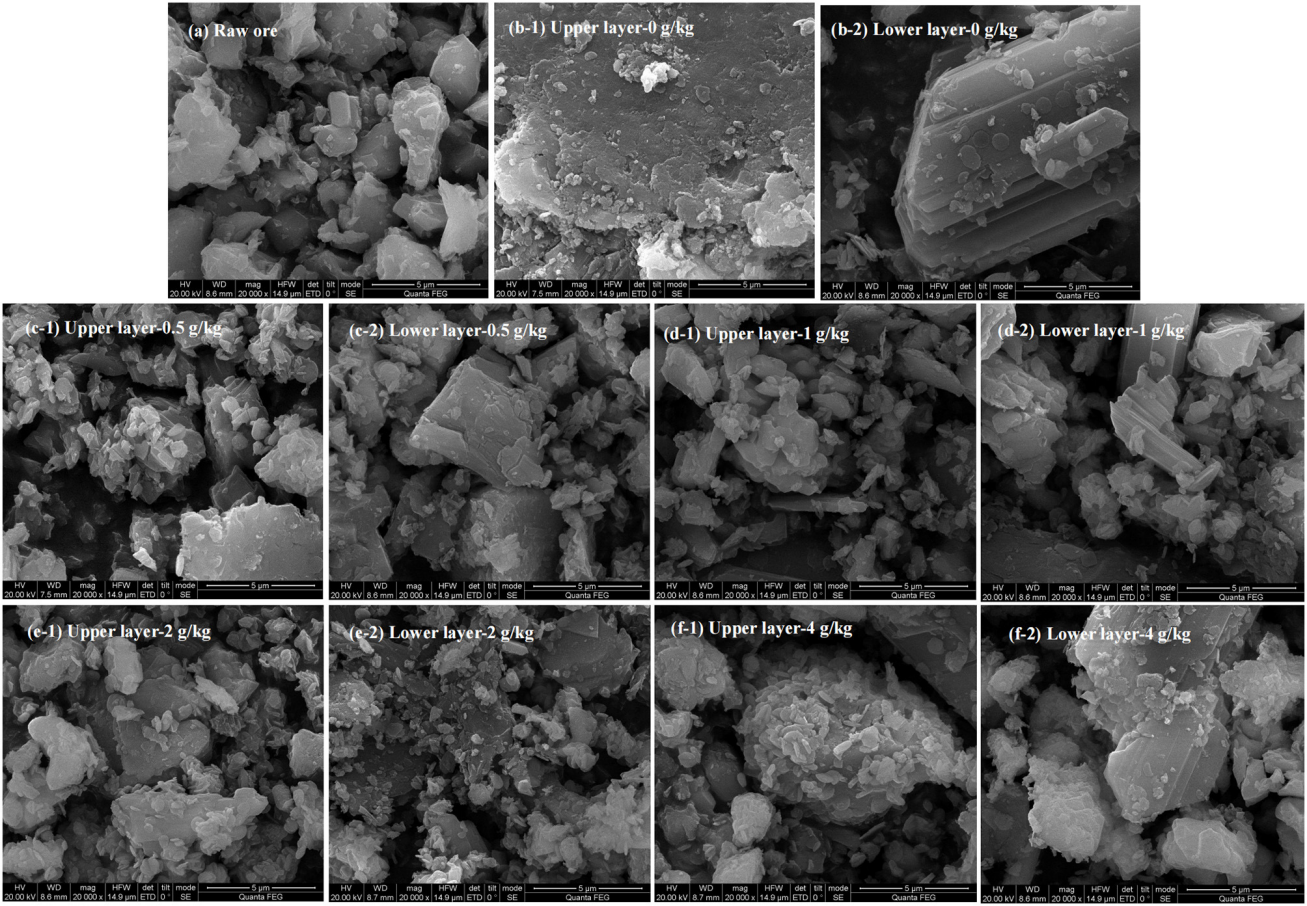
SEM analysis of raw ore and bioleaching residues in the column reactors with different sulfur dosages [**(a)** raw ore; **(b)** 0 g/kg; **(c)** 0.5 g/kg; **(d)** 1 g/kg; **(e)** 2 g/kg; **(f)** 4 g/kg].

SEM images showed a smooth surface of the raw ore (Figure 5a). A large smooth surface of the ore from control assays was also observed (Figure 5b). This indicates that the passivation layer would be generated on the ore surface. For those bioleaching residues with the sulfur enhancement of 0.5, 1, 2, and 4 g/kg, rougher surfaces with more porosity and erosion traces by bacteria were visible (Figures 5c–f). Moreover, Table 2 shows that the sulfur proportion of the ore residues in the upper and lower layer in the column was around 18% with sulfur enhancement. This was higher than that of the control. Thus, more sulfur in the residues would contribute to a better permeability of the ore layer. Consequently, a better uranium extraction was achieved. These results demonstrated that sulfur enhancement in the column reactors would strengthen the porosity of passivation layer, which could contribute to the improvement of the ore permeability and the uranium dissolution is thus promoted.

**Table 2:** Chemical components of raw ore and mineral residues in the column reactors with different sulfur dosages (0, 0.5, 1, 2, and 4 g/kg).

**Mineral components**	**Raw ore (Wt%)**	**Sulfur dosages**									
		**0 g/kg**		**0.5 g/kg**		**1 g/kg**		**2 g/kg**		**4g/kg**	
		**Upper** **layer** **(Wt%)**	**Lower** **layer** **(Wt%)**	**Upper** **layer** **(Wt%)**	**Lower** **layer** **(Wt%)**	**Upper** **layer** **(Wt%)**	**Lower** **layer** **(Wt%)**	**Upper** **layer** **(Wt%)**	**Lower** **layer** **(Wt%)**	**Upper** **layer** **(Wt%)**	**Lower** **layer** **(Wt%)**
SiO_2_	55.11	35.85	36.15	34.25	33.9	35.75	36.19	34.61	37.53	36.72	36.33
SO_3_	0.17	17.05	16.85	18.47	18.67	18.14	17.09	18.43	16.38	17.74	17.98
CaO	8.44	14.61	14.26	15.61	15.35	15.25	14.72	15.63	14.24	15.02	15.04
Al_2_O_3_	18.08	11.3	11.55	10.79	10.64	11.04	11.19	10.9	11.69	11.52	11.32
Fe_2_O_3_	2.79	9.09	9.30	9.16	9.57	8.17	8.64	9.03	8.29	7.58	7.17
Na_2_O	8.77	5.65	5.67	5.24	5.23	5.50	5.72	5.36	5.69	5.68	5.74
P_2_O_5_	4.40	4.63	4.20	4.53	4.78	4.09	4.76	4.11	4.37	3.76	4.65
K_2_O	0.34	0.827	1.06	1.03	0.97	1.09	0.782	1.00	0.838	1.01	0.769
MgO	0.91	0.42	0.408	0.37	0.36	0.375	0.351	0.388	0.414	0.398	0.421
TiO_2_	0.26	0.342	0.35	0.35	0.34	0.355	0.342	0.342	0.338	0.344	0.33
ZrO_2_	0.06	0.0614	0.0855	0.07	0.07	0.081	0.0865	0.0684	0.0775	0.0601	0.0468
ZnO	0.03	0.0864	0.0384	0.05	0.03	0.0466	0.0356	0.0445	0.0512	0.0827	0.0992
MnO	0.08	0.0279	0.0276	0.03	0.03	0.0294	0.0281	0.0306	0.0289	0.0273	0.0257
UO_2_	0.240	0.056	0.071	0.040	0.044	0.032	0.035	0.035	0.039	0.030	0.036

As shown in Figures 5b–f, the surface of the lower layer ore was much smoother than that of the upper one, indicating that a lesser extent of erosion occurred. This was also reflected by the higher uranium extraction of the upper layer. Furthermore, this observation was consistent with the lower uranium concentration in the residues (Table 2). The possible reasons for this phenomenon were as follows: firstly, the lower layer contained less oxygen than the upper layer and it endured greater pressure. These might inhibit the bacterial growth and activities (Yang et al., 2022). Moreover, bacteria turned into the decline stage at the bottom of the column reactors due to lack of nutrients in the late-bioleaching phase. The leaching process would lead to the accumulation of other metals beyond the bacterial tolerance, which was inhibitory to bacterial growth (Sasaki et al., 2009; Qiu et al., 2021). Additionally, passivation substances gradually accumulated and wrapped the ore surface at the later stage of uranium leaching. Thus, the contact between bacteria and minerals was impeded. Consequently, the leaching of uranium was restricted (Wei et al., 2020). As can been seen in Table 2, the elements Al, Na and Mn were dissolved into the leach liquor simultaneously, resulting in a decrease of the content in the leaching residues. The content of Ca, Fe and K on the residue surface was 2–3 times that of the raw ore. We infer that jarosite as the passivation layer was formed on the surface of the residues (Tuovinen and Bhatti, 1999; Yang et al., 2022). As the upper layer had higher uranium extraction, the passivation layer was most likely formed in the lower layer of the column reactors.

### 3.5. Effects of sulfur enhancement on bacterial community composition

**Figure 6:**
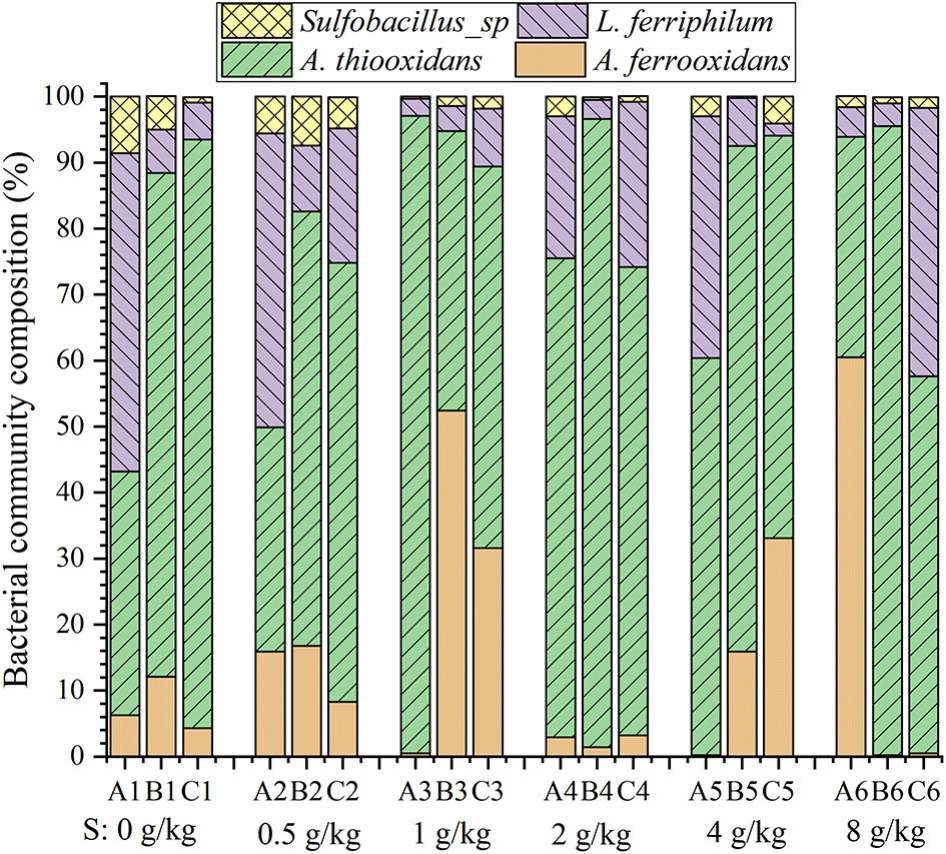
Bacterial community composition in the leach liquor **(A)** and on the ore surface of the upper **(B)** and lower **(C)** residues in the column reactors with different sulfur dosages (0, 0.5, 1, 2, and 4 g/kg).

Figure 6 shows the effect of sulfur dosages on bacterial community composition. Sulfur-oxidizing bacterium *A. thiooxidans* was the dominant population both in the leach liquor and on the ore surface in the column reactors (Figure 6). It was reported that a higher proportion of *A. thiooxidans* could metabolize complex sulfur sources and further promote the acidification of the ores in a complicated and adverse environment. In this way, the leaching efficiency was improved. The contribution of *A. thiooxidans* to the leaching efficiency was especially obvious in the initial bioleaching phase (Brune and Bayer, 2012; Li et al., 2017). It is noteworthy that the iron-oxidizers (*L. ferriphilum* and *A. ferrooxidans*) and sulfur-oxidizer *A. thiooxidans* maintained a good balance (nearly 1:1) on the ore surface at the sulfur dosage of 1 g/kg (Figure 6). These assays with 1 g/kg sulfur showed the best uranium extraction (Figure 3A). Cells of *A. ferrooxidans* exerted iron oxidation ability if both of the iron and sulfur coexisted in the substrate. Therefore, the iron-oxidizing and sulfur-oxidizing bacteria with a good population balance on the ore surface would decompose ore more effectively if presented in a favorable sulfur enhancement in the uranium column reactors.

### 3.6. Uranium bioleaching mechanism model upon sulfur enhancement

**Figure 7:**
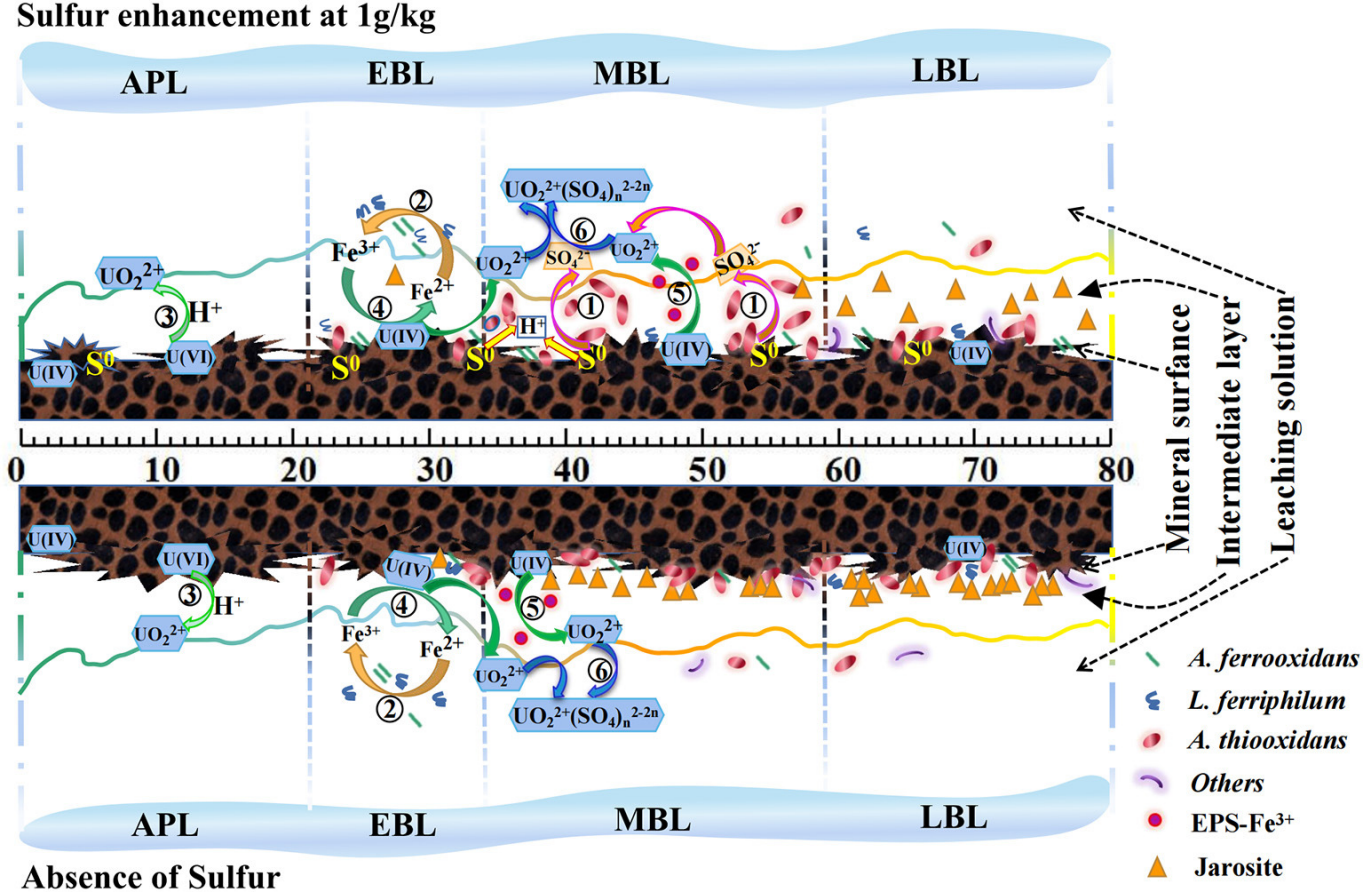
A mechanism model for uranium bioleaching with sulfur enhancement (1 g/kg) vs. the control coupling with the bacterial community and chemical reactions [APL, acid pre-leaching phase; EBL, early-bioleaching phase; MBL, mid-bioleaching phase; LBL, late-bioleaching phase. The serial number ①-⑥ represented the Reactions (1)–(6)].

To better interpret the effects of sulfur enhancement on uranium bioleaching in column reactors, a possible mechanism model (Figure 7) was proposed based on the data from the uranium dissolution reactions and kinetics, ore residue characteristics and bacterial community structures (Figure 7).

As shown in Figure 7, U (VI) in the ore could be dissolved by H^+^ attack, and the U (IV) could be dissolved by the Fe^3+^ as indirect mode or by EPS-Fe^3+^ as indirect-contact mode. Thus, the uranium extraction could be achieved at low pH and high redox potential. The low pH can be maintained by the oxidation of *A. thiooxidans* to sulfur or reduced inorganic sulfur compounds, and the high redox potential can be achieved by the oxidation of *A. ferrooxidans and L. ferriphilum* to ferrous iron. The sulfur-oxidizer *A. thiooxidans* cannot only reduce the pH in the leaching system, but also rapidly oxidize the sulfur layer on the ore surface, which was conducive to uranium bioleaching. The Fe^3+^ generated by iron-oxidizers can oxidize U (IV) to UO${}_{2}^{2+}$ by an indirect mode [Reaction (4)]. Extracellular polymeric substances (EPS) secreted by the bacteria can enrich Fe^3+^ and form EPS-Fe^3+^ complex to increase the oxidation efficiency of U (IV) to UO${}_{2}^{2+}$, namely indirect-contact mode [Reaction (5)]. Furthermore, the SO${}_{4}^{2-}$ generated by *A. thiooxidans* preferred to complex with UO${}_{2}^{2+}$ to form UO_2_ (SO_4_) ${}_{\text{n}}^{2-2\text{n}}$ in the leachate under acidic conditions (pH ≤ 4.5) [Reaction (6)], which can promote the uranium dissolution kinetics. Meanwhile, *A. thiooxidans* can change the structure of the passivation layer via sulfur oxidation activities (Li et al., 2017) and improve the permeability of the ore layer. Admittedly, excessive Fe^3+^ is likely to result in the generation of jarosite, and excessive sulfur would lead to the accumulation of elemental sulfur or polysulfide on the mineral surface in the bioleaching process. Besides, the results showed a good balance of the iron-oxidizing bacteria and sulfur-oxidizing bacteria on the ore surface would be one of the important factors for uranium bioleaching performance. Therefore, these results indicate that the quantitative balance of Fe^2+^ and sulfur, and the balance of the iron-oxidizing bacteria and sulfur-oxidizing bacteria are two of the key factors in the uranium bioleaching process. Suitable amount of sulfur addition is critical to improve the leaching kinetics.

## 4. Conclusions

The uranium extraction achieved 86.2% with proper sulfur enhancement (1 g/kg) after 77 days of bioleaching in the column. Uranium leaching was increased by 12.6% vs. the control. The uranium leaching kinetics followed an internal diffusion through product-layer controlled model. The sulfur enhancement could strengthen the porosity of passivation layer and improve the ore permeability. The sulfur enhancement at 1 g/kg could maintain a suitable balance (nearly 1:1) of the iron-oxidizers and sulfur-oxidizers on the ore surface, which is helpful to decompose the ore effectively. Quantitative balance of Fe^2+^ and sulfur, and the balance of the iron-oxidizing bacteria and sulfur-oxidizing bacteria are two key factors influencing the uranium bioleaching process. This work highlights a cost-effective alternative of uranium extraction from complex ores by proper sulfur enhancement.

## Data availability statement

The datasets presented in this study can be found in online repositories. The name of the repository and accession numbers can be found below: Genbank, NCBI; *Acidibacillus ferrooxidans*, OQ071633; *Acidithiobacillus thiooxidans*, OQ071634; *Leptospirillum ferriphilum*, OQ071635; *Sulfobacillus* sp, OQ071636 and OQ071637.

## Author contributions

QL: conceptualization, methodology, investigation, writing—review and editing, funding acquisition, and project administration. YY: formal analysis, writing—original draft, and visualization. JM: investigation and writing—original draft. JS: methodology and writing—review and editing. GL: writing—review and editing and supervision. RZ: validation, writing—review and editing, visualization, and resources. ZC and TL: validation and writing—review and editing. XL: validation and writing—editing. All authors contributed to the article and approved the submitted version.
