# Supplementary figures and images for: Sulfur enhancement effects for uranium bioleaching in column reactors from a refractory uranium ore
Source: Front Microbiol. 2023 Jan 26;14:1107649. doi: 10.3389/fmicb.2023.1107649 (PMC9911114; doi:10.3389/fmicb.2023.1107649)

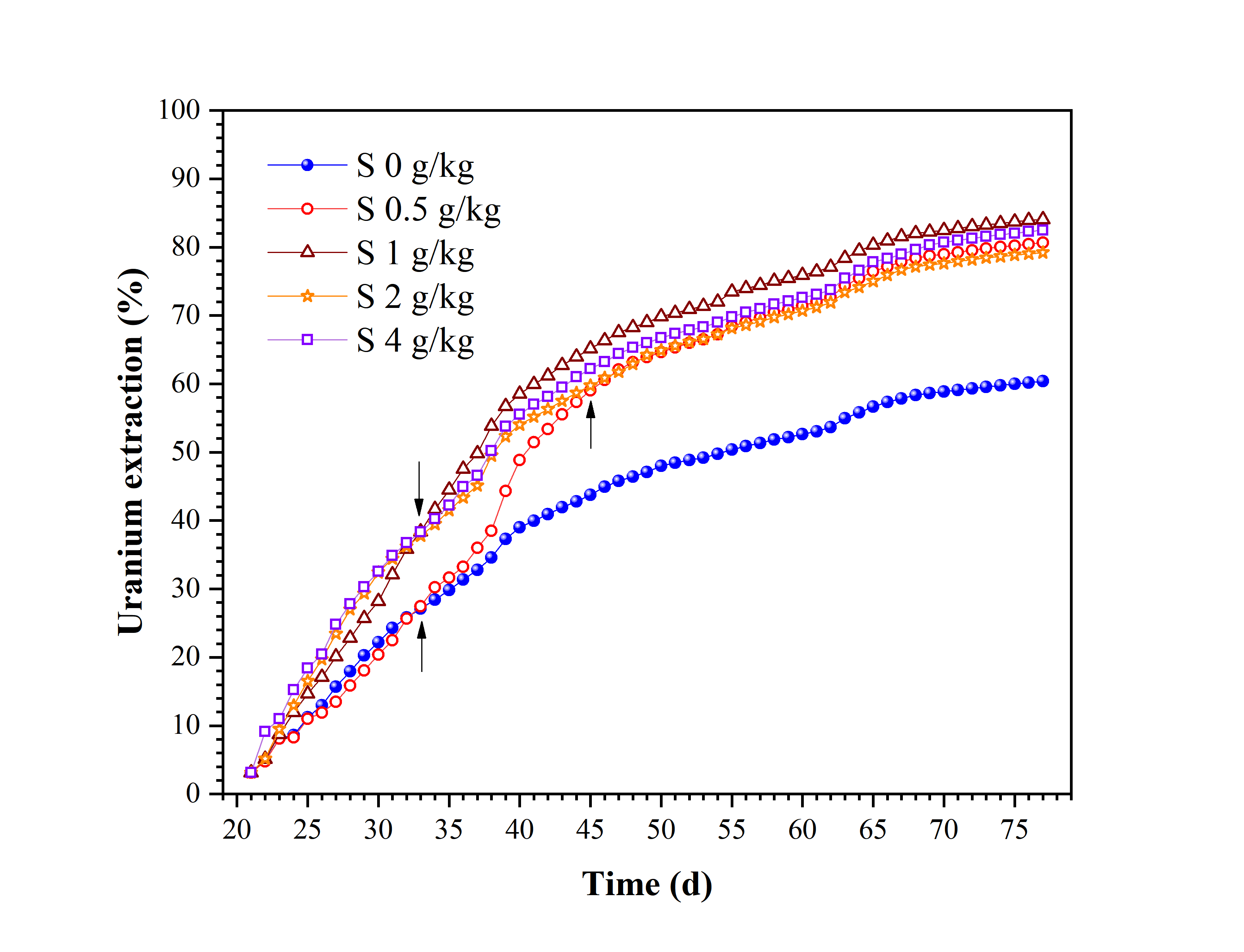

Supplement: Supplementary file 1 [file Image_1.tif]
